# Adapting the scrum framework for agile project management in science: case study of a distributed research initiative

**DOI:** 10.1016/j.heliyon.2019.e01447

**Published:** 2019-03-29

**Authors:** Enric Senabre Hidalgo

**Affiliations:** Internet Interdisciplinary Institute (Universitat Oberta de Catalunya), Av. Carl Friedrich Gauss, 5, 08860, Castelldefels, Barcelona, Spain

**Keywords:** Information science, Sociology

## Abstract

This article explores the adoption of agile methods for the management of projects in collaborative research initiatives. The use of the scrum framework, a specific set of agile principles and practices for self-organizing cross-functional teams in software development projects, is currently being expanded to other types of organizations and knowledge management processes. The study addresses the extent to which key principles and tools usually used in scrum, due to their potentially positive influence on team dynamics and efficiency, can contribute to the collaborative management and coordination of tasks in research processes. The responses from interviews with 17 researchers, as well as participant observation and analysis of online activity, are examined and presented as a case study on the adoption of scrum practices in a distributed research centre dedicated to the evaluation of public policies. Results indicate that integrating agile methods and principles for interdisciplinary collaboration requires a high degree of flexibility and a “learn by doing” approach.

## 1 Introduction

### 1.1 Team-based collaboration in research

Team-based collaboration is a critical factor in research organizations and scientific fields, as knowledge is increasingly being generated by research teams (Wuchty et al., 2007; Wagner et al., 2017). Literature on research practices indicates that teamwork and collaboration dominate knowledge production in academic organizations and is prevalent in large-scale international research networks (Cooke and Hilton, 2015). Academics and investigatory teams working on science, engineering and social science disciplines have shifted towards collective research (Wuchty et al., 2007). The benefits of research collaboration range from an increase in citations as a result of the co-authorship of papers to better use of existing resources (Ynalvez and Shrum, 2011). Other benefits include the capacity to generate wider social impact through large-scale research projects (Bammer, 2008), and more opportunities for knowledge transfer and learning (Lassi and Sonnenwald, 2010) or for managing complexity (Helbing et al., 2015).

The study of collaborative research networks from diverse perspectives has gained momentum in recent years (Wang and Hicks, 2015) because funding agencies, which prioritise better use of existing resources, prestige and international reputation, are encouraging large-scale collaborative research programs (Smykla and Zippel, 2010). In this respect, research collaboration may be viewed as a self-assembling entity, characterized by fuzzy boundaries and the tendency to function as networks (Spinuzzi, 2015; Wang and Hicks, 2015) that involve not only different research institutions, but also expand to include collaboration with industry, governments or civil society (Bridgeford and Amant, 2017). Collaboration may occur across sectors and types of organisations (Bozeman and Corley, 2004), such as government-based research programs, that usually emphasize multidisciplinary and applied research (Gray et al., 2001), or in industry, where the confines of conducting research are usually bypassed for the sake of academic publishing and the search for utility for the non-academic partners (Perkmann and Walsh, 2009).

Several authors contend that this shift to research collaboration is occurring amidst a trend towards disruptive adoption of information and communication technologies (ICT) in knowledge-intensive organizations (Jirotka et al., 2013; Borgman, 2010; Powell and Snellman, 2004). At present, collective research is undertaken in more distributed, reflexive and less hierarchical work arrangements (Zuboff, 1988), thereby expanding the possibilities for complex multidisciplinary and interdisciplinary collaborations on varying scales (König et al., 2013). In parallel to the prevailing opinion that research collaboration correlates with high productivity (Daradoumis et al., 2012) and quality results (Rigby and Edler, 2005; Liao, 2011), some scholars describe it as a difficult and ever-changing process, particularly when involving collaboration between geographically dispersed remote teams (Eccles et al., 2009). Key challenges in team-based collaborative research management relate to issues of commitment, transparency or communication and monitoring (Keraminiyage et al., 2009). Collaboration across disciplines also requires progressive adaptation of a shared language and different types of tools (Jeffrey, 2003).

Kraut et al. (1987), describing the process of collaborative research in scientific teams, explain how plans become progressively more detailed and specific, but can often be revised and even abandoned without negatively impacting collaboration. Other challenges in collaborative research management relate to the need for supervision and coordination among peers (Delfanti, 2016), or to coordinating an activity that is continually evolving (König et al., 2013). Large-scale research projects usually imply more dedication to leading and coordinating each process, from research design to the collaborative authorship of papers and reports (Bozeman and Corley, 2004). In this sense, collaborative research projects often require new project management techniques (Vom Brocke and Lippe, 2015). Methodologically, these additional complexities when performing scientific activities represent an evolving interdisciplinary field requiring various types of analysis of how and when collaborative research is implemented (Sonnenwald, 2007; Katz and Martin, 1997).

### 1.2 Agile project management

Agile project management (APM) or “agile methods” represents a team management approach and a productivity framework that supports continuous and incremental progress on work priorities, even in the face of changes. APM has its origins in the agile processes of software development, such as Scrum, XP, DSDM, Cristal, etc., which are programming methodologies based on adaptability to any change as a means to increase the chances of success of a project (Cohen et al., 2004). Most agile methods try to minimize risks during the execution of a project by developing software in iterations, which usually last from one to four weeks. Each iteration is like a miniature project of the final project, and includes all the tasks necessary to implement new functionalities: planning, requirements analysis, design, coding, testing, and documentation. An agile programming project aims to release new software at the end of each iteration, and between each iteration the team reevaluates its priorities.

APM has gained in popularity in recent years, primarily in the software industry (Scrum Alliance, 2016) but is progressively breaking into other domains (Ciric et al., 2018). In the late 1990s software development teams started to apply agile methods for the improvement of programming processes by making them more continuous and incremental on the basis of agile principles such as adaptability, personal and group autonomy, modularity and self-organized collaboration, as defined in the *Agile manifesto* (Beck et al., 2001). The manifesto was a reaction to the weaknesses and rigidity of popular plan-based software production methodologies, such as the previously highly acclaimed “waterfall” method, which has been criticised mainly for its lack of responsiveness to change (Cockburn, 2002). APM, more so than other management frameworks, emphasizes teamwork by focusing on the social aspects of software development (Rosenberg and Stephens, 2003), channelling co-creation between programmers and other participants in self-organized, cross-functional teams (Hoda et al., 2013), with collective ownership and collective responsibility as key attributes (Robinson and Sharp, 2005). According to Conforto et al. (2014) APM practices include: (1) the use of the “project vision” concept, (2) simple communication tools and processes, (3) iterative planning, (4) developing activities via self-managed and self-directed teams, and (5) frequently applying project plan monitoring and updating activities.

Despite the critique by some authors that the agile manifesto principles are insufficiently grounded in theory (Conboy and Fitzgerald, 2004) and claims that APM practices and principles lack focus on software architecture (Rosenberg and Stephens, 2003), that it is suitable for small teams but not larger projects (Cohen et al., 2004), and that it is not a panacea for effective project management (Veneziano et al., 2014), the majority of peer-reviewed papers and other empirical studies highlight the benefits of adopting agile methods (Dybå and Dingsøyr, 2008). The growing use of APM seems mainly due to the potential for optimizing the operative capacity of teamwork in short implementation cycles and the positive influence exerted on team dynamics (Fernandez and Fernandez, 2008). Some other documented benefits of the adoption of agile methods relate to the visualization and sharing of progress on tasks, thereby maximizing possibilities for success in projects in complex and multidisciplinary environments (Cao et al., 2009).

As indicated earlier in this discussion, the use of APM has expanded beyond software development to other organizational contexts (Ciric et al., 2018; Rigby et al., 2016). Analyses have been conducted on the implementation of agile management in product development (Lehnen et al., 2016; Stare, 2014), educational projects (Grimheden, 2013), construction projects (Demir and Theis, 2016), venture capital groups (Sutherland and Altman, 2009), innovation processes (Hannola et al., 2013) and the management of projects in libraries (Niemi-Grundström, 2014) and banks (Niclasen and Stoklund, 2016). In parallel to evidence of the contribution of AMP to a more flexible and responsive organizational culture outside of the software development world (Küpper, 2016), there is increasingly more academic literature on the adoption of agile methods for different types of collaborative research processes and scientific projects. For example, studies highlight the successful utilisation of APM in academia-industry collaboration (Sandberg and Crnkovic, 2017; Santos et al., 2016; Ota, 2010); the application of agile methods to faculty work (Pope-Ruark, 2017) and bridging the gap between research and practice in the management of case studies (Barroca et al., 2015). There is evidence of success in enabling collaboration in working with and mentoring PhD students (Hicks and Foster, 2010); developing prototypes in “Action Design” research projects (Keijzer-Broers and de Reuver, 2016); coordinating a large-scale European research project with distributed teams (Marchesi et al., 2007) and for the production of multidisciplinary research reports (Senabre Hidalgo, 2018). APM can also be successfully used in managing a research and development laboratory (Lima et al., 2012); adopting experimental ethnography approaches in the workplace (Mara et al., 2013); using evidence-based projects for behavioural interventions (Hekler et al., 2016); or adapting lean software development in the biopharmaceutical sector (DeWit, 2011) or in human-centred research practices (Armstrong et al., 2015).

### 1.3 The scrum framework

The scrum framework is one of the most adapted APM principles and practices (Lei et al., 2017). The scrum methodology facilitates the coordinated activity of programmers who break their work into small tasks that can be completed within fixed duration cycles or “sprints”, tracking progress and re-planning in regular meetings in order to develop products incrementally. The first reference to the term “scrum” appeared in Nonaka and Takeuchi's (1995) “The New New Product Development Game”, where it was defined as a holistic approach to flexible, autonomous and dynamic teamwork with six main characteristics, namely “built-in instability, self-organizing project teams, overlapping development phases, ‘multilearning’, subtle control, and organisational transfer of learning.”

In their study on leading technological companies in Japan and in the United States, via interviews with CEOs and engineers about how they developed successful innovative products, the authors identified those key characteristics and defined them as follows. (1) Built-in instability: when top management offers a project team a wide measure of freedom and also establishes challenging goals. (2) Self-organizing project teams: when groups take initiatives and develop an independent agenda for their work. (3) Overlapping development phases: instead of a sequential approach (where a project goes through several phases in a step-by-step fashion) the overlapping approach emphasizes speed and flexibility, and enhances shared responsibility and cooperation. (4) ‘Multilearning’: when team members engage in a continual process of trial and error, “learning by doing” along two dimensions: across multiple levels (individual, group, and institutional) and across diverse functions. (5) Subtle control: although teams can be largely on their own, management establishes checkpoints to prevent instability, ambiguity and tension, while in parallel there's also control through “peer pressure”. (6) Organisational transfer of learning: participants transfer their learning to others outside the group, creating conditions for new projects, and also by assigning key individuals to subsequent projects. Knowledge is also transmitted through the organization by converting project activities to standard practice.

Given the focus on a team's collective intelligence, the scrum framework usually requires facilitation to improve teamwork and motivation, to clarify who's doing what, to help with conflict resolution techniques, and to ensure that team members contribute (Rigby et al., 2016). Like the rest of the team, the facilitator or “Scrum Master”, who can be an experienced colleague or a professional hired for such purpose, works on a Kanban board, which is used to document the elements, as well as enable the social aspects of tasks (Sharp et al., 2009). The Scrum Master, therefore, performs the role traditionally assumed by a project manager or team leader and, in this case, is responsible for implementing scrum values and practices, as well as removing impediments (Cervone, 2011).

Subjecting each task to “development sprints” (a period of work averaging 14–20 days) is another practice that is directly related to the scrum methodology (Abrahamsson et al., 2017). Sprints, which are iterative cycles where a given project is developed or enhanced to produce new increments, are usually initiated with a planning meeting at which participants agree on a list of tasks to be performed by the end of a specified period. During the sprint, the team meets daily in short meetings called “standups” to track work progress and communicate (Friess, 2018) and, if necessary, resolve issues (Marcal et al., 2007). At the end of the sprint, a review or “retrospective” meeting is held at which the team examines developments that occurred during the sprint (Marcal et al., 2007). Interested stakeholders may also attend this meeting. Another scrum practice that is directly related to the APM framework, in this case derived from Lean production models, involves the small, regular releases of “minimum viable products”, as opposed to final, fully completed and evaluated outputs at the end of long periods (Münch et al., 2013).

Whether following the scrum methodology or more “light” and simple aspects of the APM framework, the adoption of a Kanban board is useful for its practicality and for tracking implementation on a daily basis (Anderson et al., 2012). The literal translation of Kanban, which is of Japanese origin, is “visual” (Kan) “board” (Ban). Using Kanban, work is broken down into tasks, with descriptions shown on cards or Post-It notes that are displayed on a shared board (usually with separate columns to reflect process). In this way, workflows are visible to all members of the team (Ahmad et al., 2013). Whether via physical or digital tools, the Kanban board infuses the agile development process with high visibility –providing a means of displaying the work assignments of the team, communicating priorities, making it easier to highlight bottlenecks, and helping to optimize efforts (Cocco et al., 2011). This key aspect of shared visibility and dynamism in the coordination of teamwork —a paradigm focused on doable and transparent tasks— is a basic tenet of the adoption of scrum practices in collaborative processes and organizational structures outside of the software development context (West et al., 2010).

## 2 Background

As the previous section argues, agile methods constitute an increasingly popular management process based on principles of adaptive planning, continuous improvement, frequent consultation with participants and small and regular releases (Cao et al., 2009), as well as simplicity and dynamism (Abrahamsson et al., 2017). In this paper —an exploratory analysis— the focus is on the appropriation of scrum as a methodological framework and its experimental use in the management of distributed and interdisciplinary research initiatives, with the aim of identifying the experiences and perceptions of researchers in the adoption of APM principles and practices, as well as the potential benefits and limitations.

In this regard, the paper seeks to answer the following research questions:

• Which conditions favour the appropriation of APM for research collaboration?
• To what extent can specific scrum principles and tools be adopted in interdisciplinary contexts?
• What are the limitations and advantages of adapting agile methods in a distributed research organisation?

The UK-based Centre for the Evaluation of Complexity Across the Nexus (CECAN, cecan.ac.uk) is the focus of this case study. CECAN, a research centre hosted by the University of Surrey, was established in 2016 and comprises more than 50 members working in 14 different academic organisations such as the University of Warwick, the University of York, Cranfield University and Newcastle University. Conceived as a network of social scientists, policy makers, policy analysts and experts, CECAN explores, tests and promotes innovative policy evaluation approaches and methods pertaining to food, energy, water and the environment across nexus domains. The organisation carries out this mission through the implementation of a series of ‘real-life’ case study projects with UK partner institutions including the Economic and Social Research Council (ESRC), the Natural Environment Research Council (NERC), the Department for Environment Food and Rural Affairs (DEFRA), and the Department for Business, Energy and Industrial Strategy (BEIS), among others.

CECAN teams develop case studies and other interdisciplinary initiatives around research methodologies, complex systems, policy evaluation (in areas related to sustainability or economic promotion), as well as new evaluation and assessment methods. As a distributed initiative incorporating experts from diverse knowledge areas with varying levels of dedication and time capacity for projects, and in the absence of a central physical office or shared space, it required a specific approach to coordination and management. For this purpose, from its early operations, CECAN adopted some APM principles and practices derived from the scrum framework, as well as a digital Kanban board for managing the information and knowledge generated by its teams.

## 3 Methods

This case study utilised three methodological approaches and data sources: participant observation, analysis of online activity and semi-structured interviews. This combination of approaches forms the basis for the analysis of the adoption of agile principles and practices and the scrum framework at CECAN. A six-month period of participant observation of various activities hosted by CECAN resulted in the generation of a database of observation notes. The notes covered team dynamics and references to APM principles and practices in four meetings and two workshops, as well as the direct experience of facilitating an agile process for a specific project with four participants from CECAN. The observation notes and direct experience, together with the parallel literature review on agile principles and the adoption of agile practices in a variety of contexts, served as the basis for the development of the structure and areas of analytical focus.

The statistical and content analysis involved group interactions on the digital Kanban tool Trello (trello.com). Trello, a web-based project management application, is used as the main channel for coordination and knowledge sharing at CECAN. Data gathered by exporting JSON files and manual scraping of web content from 43 Trello boards facilitated the understanding of patterns of interaction between levels of activity and types of interaction. More specifically, to observe the correlation between the number of active participants, topics covered on each board and relevant actions on cards (change of status, comments and attachments) were analysed. This provided an overview of relevant interactions as well as active projects related to the centre, and allowed for more detailed coverage of the use of digital Kanban boards, which was one of the topics addressed by the interview questions and the data analysis.

An interview protocol, designed as the third and main source of data for the study, was used for seventeen semi-structured interviews with researchers (nine men and eight women) from diverse disciplines and institutions who have experience with the adoption of agile practices in their projects (Table 1). The interview questions were developed with the goal of obtaining different perspectives on the experiences of researchers with the use of agile methods for collaboration in their projects. Using the semi-structured approach, the interviews took the form of conversations guided by questions on APM practices, the scrum framework, teamwork and research activity, which naturally evolved through relevant threads of conversation. The participants varied by field, academic background and experience; some were early-career while others were mid- to late-career. Ten researchers (RC), from several universities and backgrounds who collaborate with CECAN on regular basis, were interviewed. Among them, six interviewees had the specific role of Scrum Master at CECAN, with responsibility for the coordination of various case studies, on which other researchers and stakeholders from various institutions collaborate. The remaining seven interviewees (RE) were researchers and practitioners affiliated with institutions outside CECAN, who also had direct experience in the application of agile principles, to some extent, in research or academic-related projects. These seven additional interviews were conducted in the same period as the other ten, and served as a control group for contrasting diverse observations and for understanding widely important issues derived from interviews to CECAN members.

**Table 1:** Researchers and agile practitioners interviewed.

Role of interviewee	Institution	Gender	Involvement with CECAN	Scrum Master role
Associate Professor	University of Warwick	Female	Yes	Yes
Research Associate	Newcastle University	Female	Yes	Yes
Research Fellow	University of Westminster	Male	Yes	Yes
Research Associate	Newcastle University	Female	Yes	Yes
Research Director	University of Surrey	Male	Yes	No
Postdoctoral Researcher	University of York	Male	Yes	Yes
Research Fellow	University of Westminster	Female	Yes	Yes
Research Director	Newcastle University	Male	Yes	No
Research Director	University of Westminster	Male	Yes	No
Senior Consultant	Risk Solutions	Female	Yes	No
Senior Researcher	Technical University of Denmark	Female	No	No
Associated lecturer	Open University of Catalonia	Female	No	No
Research Professor	Open University of Catalonia	Male	No	No
Researcher	Open University of Catalonia	Male	No	No
Co-Founder	Collaborative Knowledge Foundation	Male	No	No
Chief Experience Officer	BeyondCurious	Female	No	No
Consultant	Risk Solutions	Male	No	No

To capture interview data accurately, each interview (which lasted approximately one hour per participant) was audio-recorded and later transcribed for coding. Using a grounded theory approach, data was coded for emerging themes (Martin and Turner, 1986). Themes were discovered through a recursive coding process, then grouped into three areas of inquiry related to the research questions (Table 2): (1) conditions for adopting agile methods in research, (2) adoption of scrum practices and tools, and (3) limitations and advantages of APM adoption in a distributed research organisation. Results were collated into a structured corpus of voices following that sequence, with the most representative and relevant answers selected from interviewees.

**Table 2:** Themes derived from interviews in relation to research questions.

Areas related to research questions	Themes
Conditions for adopting agile methods in research	Complex and changing setting
	Capacity for self-organisation
	Flexibility
	Adaptivity
Adoption of scrum practices and tools	Facilitation roles (Scrum Masters)
	Kanban boards
	Development sprints
	Incremental development
Challenges for APM adoption in a distributed research organisation	Need for balance
	Offline vs online context
	Proliferation of kanban boards
	Trust in relationships
	Types of research
	Time and resources
	*Ad hoc* adoption
	Institutional culture

The results elaborate the relationship between key principles and practices derived from the literature review on agile methods and principles and reflect the findings based on activity and perceptions of participants, while at the same time integrating a description of the basic features of the scrum framework adapted during its experimental adoption.

In relation to the interviews conducted for this study, although the method as applied in this case does not require an ethical committee approval from the author's institution (Universitat Oberta de Catalunya), all the interviewees volunteered for it and signed a consent for participation accordingly, guaranteeing that confidentiality as a participant will remain secure, and that subsequent uses of records and data will be subject to standard data use policies which protect the anonymity of individuals and institutions.

## 4 Results

### 4.1 Conditions for adopting agile methods in research

From the observations and interviews conducted with CECAN researchers, from the outset, it appears that the underlying rationale for selection was premised on key features of the scrum framework and agile methodology such as flexibility, autonomy and self-organisation.

#### 4.1.1 Complex and changing setting

Since the early operations of CECAN in 2016, its executive board promoted the idea of adopting scrum methods as a possible solution for the self-management of projects, from case studies or workshop organisation to other publication-oriented initiatives. The complexity of conducting research with groups of stakeholders who operate under existing policies, while also setting an evaluation framework for new ones, demonstrates, as one participant observed, that “unpredictable events can come along and change the system potentially” (RC1). This need to regularly adapt activity to a complex context, in a new research institution with more than 30 researchers involved (most of them part-time, and usually collaborating from a range of institutions), also presented a significant management challenge, where it seemed “quite hard for any individual to regularly keep up with all that's going on” (RC2).

#### 4.1.2 Capacity for self-organisation

Another key agile principle relates to the focus on the interactions of self-organised teams. In this case, the scrum framework facilitated regular interaction and feedback among participants. The adoption of the scrum framework was based on the same logic of self-organisation of CECAN, with teams assembled according to the interest or potential contribution of each participant to specific topics, with a logic of combining diverse disciplines and points of view. In this way, as one participant noted, “the vision comes from everyone and it is not like that one person got the direction, it actually emerges from the collective expertise of the group” (RC3).

From the perspective of participants in CECAN case studies, self-motivation was a key factor in many of the parallel projects of the centre, which usually started with very open internal calls:

The initial asking of people who wanted to be involved had to be very open, anyone who thinks they want to help is welcome to. So, I'd have that as a founding principle (RC4).

In this sense, challenges in self-organising, and especially self-assignment in adopting scrum methods for knowledge-based tasks, were cited by other researchers and professionals outside of CECAN.

An ideal scrum team is that which can sit together for a long time and listen to each other. This can significantly augment your learning process. But my theory about research is that you usually don't have this kind of team (RE1).

#### 4.1.3 Flexibility

From observations and interviews with CECAN researchers, the flexibility of the scrum framework seemed to be one of the main reasons why APM principles were considered useful and put into practice: “I felt that this was a way of rationalizing the process that we were already doing and getting it a little bit more structural, while still valuing the flexibility that we had” (RC7).

When interviewed, researchers from outside of CECAN, who have experienced the use of agile practices in academic and research settings, also considered the extent to which it is important to be flexible and start by adapting only some of the scrum principles (to avoid excessive rigidity in its application):

If you take scrum very literal it might not work. For example, if you have divided the project into small areas and manage each one with scrum then it might be very difficult to have four daily meetings in four different groups is an hour of work every day. (RE1).

#### 4.1.4 Adaptivity

Many participants viewed the agile framework as an interesting alternative, and a clear, easy concept to communicate and agree on. It is noteworthy that this occurred in the context of an organisation that deals regularly with the analysis and implementation of methodological approaches in areas of research and evaluation, adapting to different institutional environments and ways of working.

When you use the word ‘agile’, I think people don't question it. I think in a natural language sense, in an English sense, the meaning of the word has relevance and it sounds fine. If you say ‘we're going to work in an agile way’, I think that communicates quickly the idea (RC4).

Researchers interviewed from outside of CECAN also highlighted the importance of “learn by doing” during the initial adaptation of the scrum methodology to their specific domains, realising that it meant a way of approaching management by progressively trying things out:

We were already working with an agile approach but we had not called it ‘agile’. Later on, we started formalizing things and picking up more and more scrum tools and techniques to improve the ways we manage our projects (RE2).

### 4.2 Adoption of scrum principles and tools

One of the fundamental principles of the self-organising, small operative teams at CECAN was to be innovative at the management level to gain efficiency in collaboration.

We needed to adopt an approach where you can have a consensual decision making that's not necessarily a top down process, but more of a bottom up process of dialogue of mutual interaction (RC6).

It is also important to highlight that CECAN's approach to the adoption of the scrum framework was not based on specific, dedicated training or an expert coach hired for the task. It was instead based more on an evolving interpretation of the APM principles and on experimentation on the basis of an explorative, self-taught approach to the concept.

It was according to what was required and people's individual availability and restraints, and managing that set of interactions. Evidently, we were at each stage constantly thinking about agile. ‘We might have to do this and this. That's what we should do’ (RC6).

#### 4.2.1 Facilitation roles (Scrum masters)

**Fig. 1:**
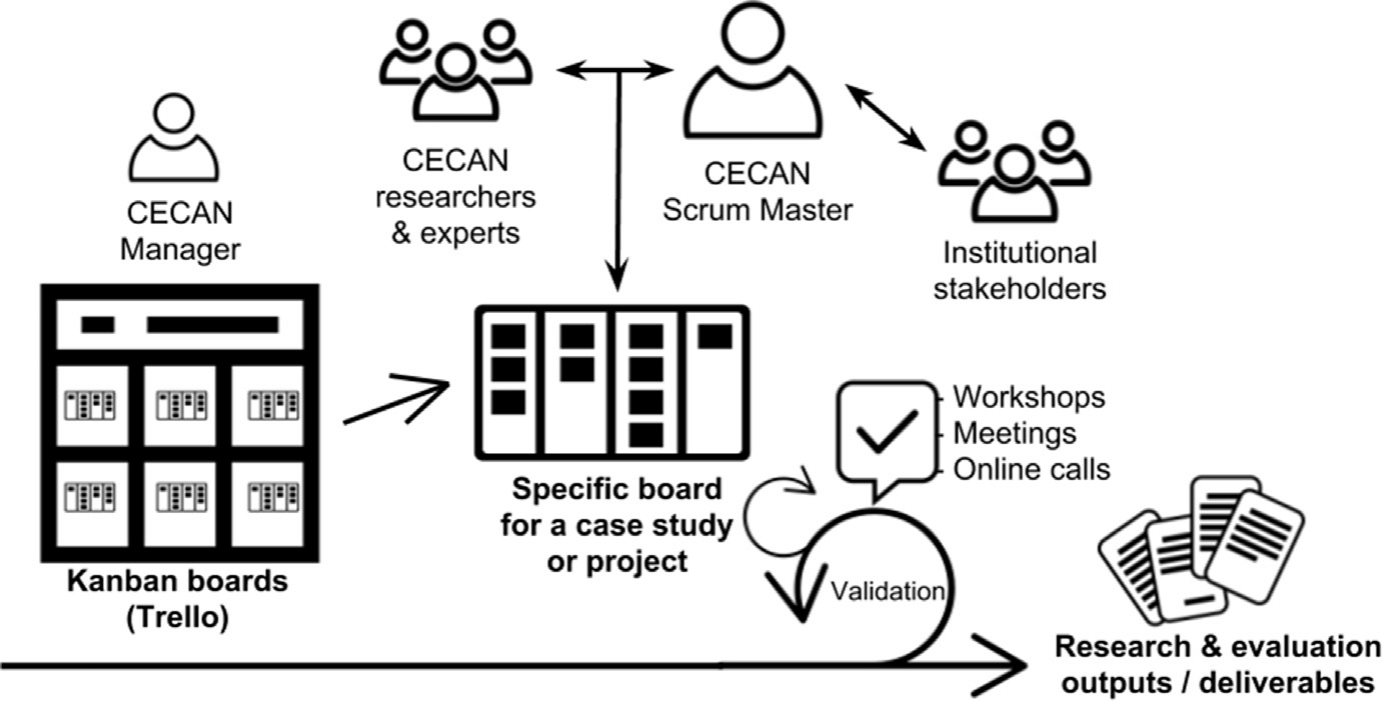
Diagram of the scrum adaptation for research and evaluation projects at CECAN.

Soon after that initial meeting at which the core principles of the scrum framework were introduced, several of the researchers collaborating with CECAN started to adopt some of its key elements. The role of Scrum Master was one of the principles adopted. At CECAN, the role was conceived as a coordinator for case studies, which had on average four, but up to eight participants (Fig. 1). CECAN Scrum Masters viewed their role as the link between specific tasks and objectives and other collaborator researchers, as well as the liaison with policymakers and representatives from government agencies. This key role was performed by CECAN researchers instead of professional Scrum Masters, and was focused on coordination, facilitating connections and providing guidelines for specific case studies.

At CECAN, Scrum Masters were seen as connectors of expertise and coordinators guided by shared goals, as one participant explained, “I see the role of Scrum Master as a kind of intermediary in an interdisciplinary project like this one. The expertise that a Scrum Master acquires is in linking an expert to an expert and that itself requires [a] particular set of expertise for CECAN, this is an ongoing challenge” (RC9).

In this sense, the role of Scrum Master could be considered an appropriation and reinterpretation. It was one of the key agile practices adopted at CECAN, and although perceived by some participants as not fully implemented, the Scrum Master seemed to play a critical facilitation role and contributed to expanding agile management practices to the various research initiatives and projects. As corroborated by the following comment from one participant, the facilitation role provided participants with transparency and guidance, as opposed to a command and control approach, as they engaged in joint activities.

The role is very much one of a leading rather than controlling. That has to be the case because there's actually quite a lot of skill involved in managing a group of researchers for whom you have … to align management responsibility. We are a consortium of fourteen different academic organisations. If I wanted to tell you or anybody else in the team ‘you have to do this, … because I'm telling you to’ they will just go away (RC2).

Considering the high volume of case studies, publications and other tasks related to CECAN activity, for researchers acting as Scrum Masters there was also the opportunity to learn from colleagues doing the same, or even to share the role:

[In a specific project] There's really two of us acting as Scrum Master because we're covering a broad complex area of policy, to which both of us bring complementary experience. So, he and me communicate, I would say, daily. With other colleagues in CECAN, usually it's once a week at least (RC1).

It is also significant the extent to which the responsibility of having a facilitation and coordination role required additional networking efforts and expertise from researchers new to the concept:

[The Scrum Master role] It was slow to develop initially. I think it was partly about building trust and establishing relationships with the policy partners, and deciding what they wanted out of the process, and really getting a grip of what they wanted to do, how they wanted to work with CECAN (RC10).

#### 4.2.2 Kanban boards

**Fig. 2:**
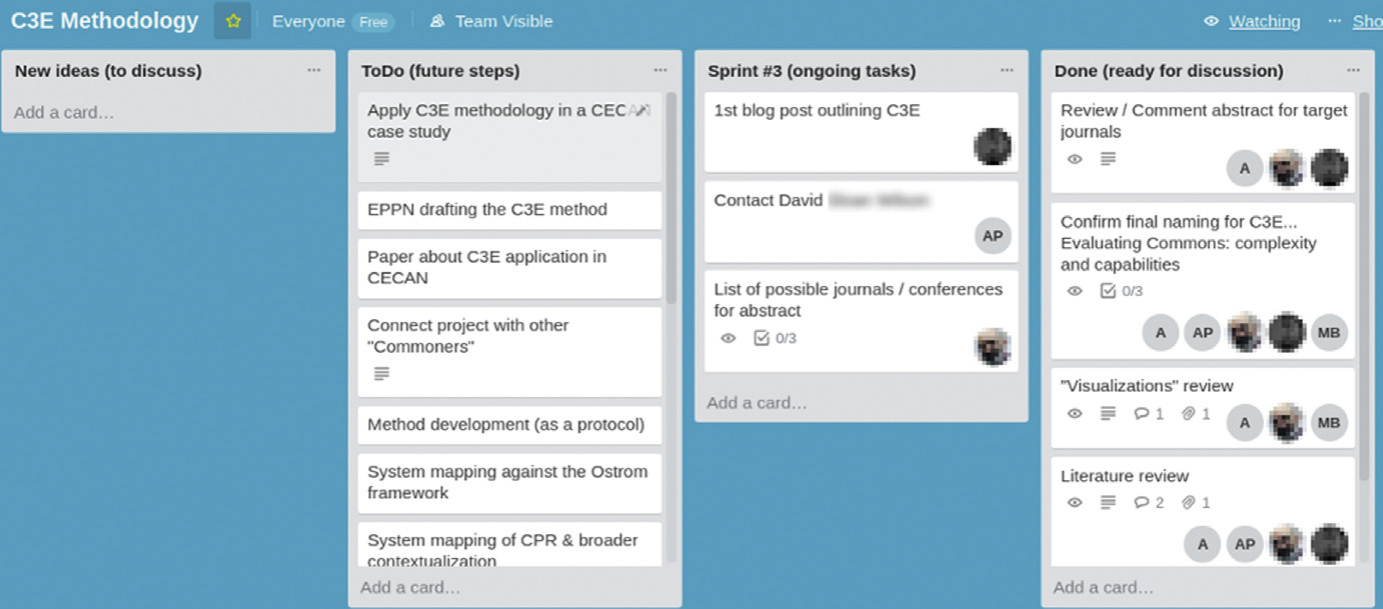
Screenshot of one of the CECAN Trello boards, with different tasks on cards.

At CECAN—a “distributed virtual organization, with so many people doing so many things with different time involvement” (RC5)—the Kanban board was one of the agile management practices adopted. The CECAN boards were digital and created using Trello, a web-based project management application, in a format replicating Post-It notes (Fig. 2). The Trello boards were one of the main channels of documentation for the centre. They were managed mainly by the Scrum Masters, and were accessed by the other CECAN researchers and occasionally by external collaborators or other stakeholders.

As explained in the excerpt below, each new initiative or discussion was eventually translated into modular pieces of information. This represents a novel way of accessing updated and valuable knowledge for the entire organisation about the progress of projects.

One of the ways that CECAN is trying to adopt an agile approach was to set up the use of Trello boards and the use of Trello as a system for those who were engaging in case studies, but also those who were engaging in non-case study activities. To update not just their own group, but the rest of CECAN as well. The use of Trello was a way of leading the case studies, updating data for example with case study notes, and what was happening on the case studies, and any particular event that was going on (RC6).

**Fig. 3:**
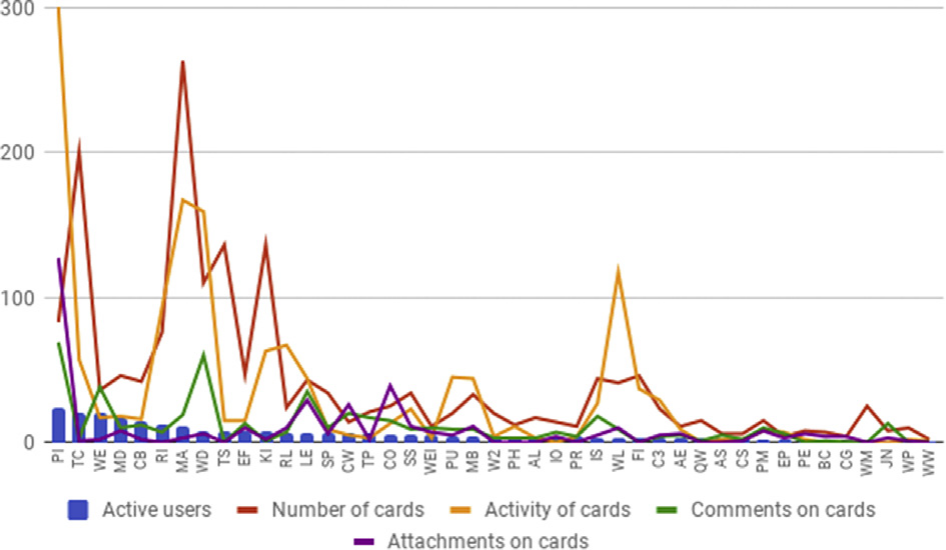
Statistic of cards on each Trello board, ordered by number of active users.

The results from a basic statistical analysis of communication and interactions on the various Trello boards at CECAN (Fig. 3) suggest that there is a relative correlation between more active users on each board, the number of cards assigned to participants and activity related to assigned cards (usually displacing them on the board according to workflows, or content edits). There appeared to be no correlation with publishing comments on cards and attaching documents to cards, as this occurred less regularly, apart from some exceptions. This would confirm that the Trello tool was used consistently through the different boards and related projects, following the typical APM process for visualizing workflows. On average, however, the analysis of the aggregated data shows that only a minority of researchers were active on the Trello platform (despite the entire organisation having full access to all the boards), which represents an unequal distribution of participation.

A comparison of the most active Trello boards, an analysis of the different levels of engagement with the tool, as well as an observation of the progressive familiarization with its functionalities and connection to APM principles, revealed that participants generally viewed their experience in using the Trello boards as an evolving process parallel to the levels of intensity and activity in the organisation. This observation is supported by the following statement from a participant who was less active in interacting or generating cards, but benefited from accessing the Trello boards: “It has proved a useful kind of map of how the case studies have evolved, sort of a narrative, if you like, a narrative of kind of key points within each of the case studies and how they developed,” (RC10).

Trello was generally perceived as practical and aligned with the need to specify, visualize and assign tasks for case studies or publications, and also “useful to have a quick overview of what is happening, and to understand what other people are doing in a quick way” (RC4). However, it represented a way of working and adapting to a specific type of interface with a significant learning curve, to which not all researchers found it easy to adapt:

I have done a lot of different projects within different project management and communication tools and it becomes too complicated in my mind (RC9).

This coincides with experiences from other researchers, and the following excerpt highlights that some colleagues, perhaps on the basis of their digital literacy, perceive this type of tool as a barrier:

There was a senior researcher struggling through it and ended up in chaos. She did not want to adapt to these things. If I said ‘Put this on the Trello board, we do not need to implement the whole thing, we can manage with something’ her response would be like, ‘Oh! What is this? I do not want to install this. I do not want to join this, it's complicated’ (RE1).

#### 4.2.3 Development sprints

With respect to the adoption of scrum methods at CECAN, the concept of sprints was less explicit or used among participants. For example, the practice of establishing regular “standup” meetings, or retrospective meetings at the end of each sprint period was not routinely followed. Instead, researchers usually established collective agreements about the duration and responsibilities related to specific tasks, depending on the project.

Like other key aspects of agile management practices, this sprint principle —although not used with the same rigour as in software development contexts— was progressively incorporated into the logic of shared communication at CECAN:

All of those things require structured communication baseline and tasks and milestone baseline. The point is not only moving forward but also ensuring that we are still understanding each other with constant feedback (RC3).

However, from some comments emanating from the interviews, the sprint also seems to be a problematic concept to appropriate from software development processes and to adopt for the peculiarity of research tasks:

Usually two weeks long, I think everyone would agree that this is how long a sprint should be. I found it funny because probably in the tech world it works, but when you have a different type of tasks the two-week period is a bit arbitrary. In one of our case studies I had workshops which were organized about one month in advance, which made a very good sense of working in sprints (RC7).

When compared with other practitioners and researchers with longer experience using agile methods in non-software contexts, there seems to be a significant difference in the way sprints were adopted at CECAN and how they were experienced in other cases, where they constituted a central part of the process:

You just don't do one sprint, it is more like doing sprints after sprints after sprints. By doing that and looking at things in many different ways, we get incredible depth (RE3).

Sometimes we block entire evenings without any other task, or plan one-day trips to finish an article with another author. Then 10 hours working and although the article is not over, it is properly drafted (RE4).

#### 4.2.4 Incremental development

It should also be noted that the concept of incremental development by small and regular releases (derived from the Lean principle of “minimum viable products”), when initially adopted from software development, was perceived as another complex approach to be tackled in the context of academic research.

We do work considering minimum viable products in a way, by thinking about stages of our work. For example, from a case study to a paper, and all the steps in between. But we don't use these exact words, is more implicit than explicit (RC5).

However, this practice, once adopted, served as an inspiration or key principle for some participants. As with the principle of sprints, some researchers developed an understanding and progressive process of adaptation of the concept of incremental research results, particularly in relation to the other organisations and stakeholders with whom they collaborated. As two participants observed:

What became quite useful I think in the use of the agile approach with [Stakeholder organisation A] was to say ‘we're going to iterate. We know right from the beginning there's going to be a lot of iterations'. To be able to describe that to them at the beginning. They never expected a final product suddenly to appear out of nowhere (RC4).

We start out with a set of objectives, but we have to adapt along the way. The set of objectives might change or how we meet those objectives might need to change, following the idea of continuous and feedback loops. That's how we're working in collaboration with [Stakeholder organisation B], but we're also having to touch base with them on a regular basis, because things are changing (RC1).

Outside of CECAN, other researchers also expressed familiarity with the principle, with some researchers even adopting and adapting the concept for use in their own research findings and academic writing.

‘Minimal viable finding’ is related to the way we are able to focus our research in every two weeks timeframes. ‘Here are things which are more promising and we are going to focus in this process’. We usually find several things but this is about highlighting something which we are going to promise and are going to deliver (RE3).

### 4.3 Challenges of APM adoption in a distributed research organisation

#### 4.3.1 Need for balance

Given the special nature of research activity, and the need for flexibility in terms of allowing experts to add value through their knowledge and expertise, there was a constant effort at CECAN to balance, adaptively, the need to produce results and to advance in the evaluation of policies without “having a hierarchical [structure of] control” (RC2). In this sense, some participants perceived the achievement of this balance as one of the most challenging aspects of assimilating new coordination approaches, and the need of leadership through the Scrum framework as a key factor for providing results without sacrificing autonomy. For some participants, when compared with the expectations implied by APM self-organisation, these attempts appeared to be not always successful.

#### 4.3.2 Offline vs online context

It is also important to highlight at this stage that there are few opportunities, in the context in which CECAN operates as a distributed research initiative, to meet offline in face-to-face meetings, with the result that facets such as self-organisation become more complicated (and usually require varying levels of online interaction), as described by this respondent:

I feel the biggest challenges with me while trying to do agile and scrum with CECAN is that we are remote, so it is difficult to have the immediate emergency or urgency of something that I need to do, compared to if you are seeing someone in person (RC7).

Limitations in team size and difficulties in adapting online because of individual research styles were also viewed as key issues that need to be resolved for agile methods to function effectively in this context.

[A specific publication project] started with probably 12 people who were interested but it was very difficult to get momentum of any kind. Everyone was interested in being involved but there was no momentum to start doing anything. So, in discussion with A., we decided to make the group much smaller to just three members. After this change, we have been working smoothly (RC7).

#### 4.3.3 Proliferation of kanban boards

Due to the initial recommendations on the use of Trello at CECAN, the boards were used for the management of various types of projects, and not only case studies for publication but also for planning of workshops, the design of new methodologies or the evaluation of policies. As a result, there seemed to be a proliferation of boards, which were not always useful or used in accordance with agile principles. One participant expressed the following view:

I think that Trello works best where people have defined responsibilities for a board and know who to contact, plus have predefined rules which are particular to a board. Probably, this concept has not been as clear to the users as it could have been, partially due to the fact that it is a new concept for everyone (RC7).

The experiences recounted by participants on the use of Trello boards as a discussion channel reflect their expectations about the tool in relation to their communication needs, given the complexity and limits of interchanging knowledge from their individual locations and institutions. Others highlighted the difficulty of adopting new digital tools instead of developing new strategies focused on the physical context.

#### 4.3.4 Trust in relationships

The high volume of case studies, publications and other tasks reflected on the numerous Trello boards, afforded researchers acting as Scrum Masters the opportunity to learn from colleagues in similar positions, or for sharing the role, and thereby learn about the implications of managing case studies as Scrum Masters in a more networked and interactive way. However, as far as the responsibility of the facilitation and coordination role is concerned, as one participant explained, the extent to which it required additional effort and progressive ‘learn-by-doing’ expertise from researchers new to the concept is significant.

It was slow to develop initially. I think it was partly about building trust and establishing relationships with the policy partners, and deciding what they wanted out of the process, and really getting a grip of what they wanted to do, how they wanted to work with CECAN (RC10)

This view is similar to that of other researchers who experienced the same challenges in similar roles in research-oriented or academic contexts other than CECAN.

#### 4.3.5 Types of research

In some of the interviews, there was often a return to the question about the extent to which it is possible to adopt agile principles in all types of research or whether, as in the view of some CECAN participants, APM principles represent a methodological framework that is more suitable to applied research and contexts where time constraints and pressure from stakeholders make it more applicable and imperative.

There are projects which are quite theoretical, with basic research, where this kind of agile is probably not likely to be very helpful. So, I wouldn't want to force agile on every piece of research (RC2).

In contrast, confirming interest in the scrum framework from a wider perspective, in front of the same question other interviewees commented how APM practices were incorporated into their own research organisations, outside of their collaboration with CECAN:

I have seen it working nicely across a variety of domains. In my small department we started it from zero, we have been doing it and have witnessed it progress. Now, we are about ten people (RC3).

I am working with two other people on projects who are not part of the original CECAN team, they have been subcontracted to come in and help work on it. I have been ‘scrumming’ with them offline, not using the traditional forums like Trello (RC7).

#### 4.3.6 *Ad hoc* adoption

However, other perspectives also addressed the complexity of applying agile principles to CECAN research and evaluation outputs and the key limitations of time and resources, as well as its correlation with the need for more flexibility in coordination:

I think adopting an agile approach in a prescriptive way it's not necessarily effective. It's easy to be quite agile in the sense of having a very weekly sense of meetings, at a particular time on a particular day, if the people that are involved are not overly constrained in terms of time or labour or any sort of resource constraints. When they are, then you have to be quite adaptable or flexible according to the regularity, according to the main principal parties involved. So, I think from that perspective, more open agile use approach is perhaps more effective than a prescript one that says ‘we're going to be this regular in terms of when our meetings are going to happen, when we need to update the Trello board, so on and so forth’ (RC6).

In this respect, other experts with experience in the utilisation of agile principles outside of CECAN also emphasized the importance of flexibility and openness when adopting these methodologies, instead of following blindly the rules and proceedings as they are established in software development processes.

I have come up with methodologies, and I know that they're all made up, there are frictional of context specific tricks, and methods, and tools and thinking. Agile presupposes that the ‘big box’ methodologies can ignore context in a way. Like a call and response mechanism which is very rule based and explicit, and I don't think that this is how a method works (RE5).

#### 4.3.7 Institutional culture

For other researchers, who are familiar with scrum and agile methods, another key issue is related to the complexity and the management challenges embedded at the institutional level in universities and scientific departments:

The group can be agile but it faces a system like the academia and the university that is not agile. So, the motivation to do research and at the same time adapt to new ways of doing is complicated to manage (RE4).

Managers of research projects and IPs are not trained in project management, nor these skills are covered in PhD courses or similar. You can only self-learn about it, or explore on your own your ability to do so by acquiring collaboration skills and techniques (RE7).

## 5 Discussion

The objective of this article was to explore the adoption of agile methods in a distributed research initiative, and especially the appropriation of the scrum framework as a coordination and communication solution for the management of collaborative interdisciplinary projects. Taking into account the specific characteristics applicable to research in academic and scientific areas (as a separate context from software development processes, where the APM framework was developed and is widely used), the adoption seemed successful overall in that it facilitated the generation of new dynamics of collaboration, benefiting from some APM principles and practices in various ways. However, the process was also challenging and had some limitations in terms of a shared understanding and coherent application of the scrum framework, when compared to similar experiences in the use of agile methods in research projects.

In this regard, according to the data obtained from interviews, the adoption of agile methods in research collaboration is suited to organisations embedded in complex and changing settings, with some capacity for self-organisation, flexibility and adaptivity to new management approaches, which connects with the description of organizational networks (Spinuzzi, 2015, p. 58). On the other hand, relevant challenges identified for APM adoption in research point to issues related to: (1) the needed balance between efficiency and autonomy of participants, (2) the limitations of the online context for coordinating activity, (3) the tendency to proliferation of kanban boards; (4) the need to build trust in relationships when coordinating, (5) the type of research activity carried out, (6) time and resources constraints, (7) the importance of tailoring scrum principles to activities, and (8) the institutional culture of academic and research organisations.

Integrating agile methods and practices for interdisciplinary collaboration requires high degrees of flexibility and “learn by doing” approaches, similar to other project management methodologies and approaches (Lauren, 2018, p. 30). In this sense, the scrum framework constitutes a methodological framework that can be counterproductive if it is too ambitiously or rigidly implemented in this type of context, as indicated in the literature on the utilisation APM outside of the software development sector (Ciric et al., 2018). According to Nonaka and Takeuchi (1995), this type of participative management can be favourable for several types of agile development where conditions such as “built-in instability, self-organizing project teams, overlapping development phases, ‘multilearning’, subtle control, and organizational transfer of learning”, converge and are present to some extent in the philosophy of the collaboration initiative. When adopted by academic participants and experts familiar with research or evaluation methods, the scrum framework seems to be an easy concept to transfer and experiment with, even though specific tailoring to the idiosyncrasies of collaboration and personal motivations may be required when adapting APM (Gandomani et al., 2014). Also, as attested by the literature on agile software development, characteristics such as team size and specificities such as the online tools required for operating in distributed contexts seem critical, as well as its suitability for small groups but not for large projects (Cohen et al., 2004), or the significant complexity that may be experienced when adopted by remote as opposed to collocated project teams (Paasivaara et al., 2009; Teasley et al., 2000).

Scrum principles adopted by various research teams, as analysed in this study, were seen as a valuable addition to the coordination of projects, with diverse levels of agreement about their successful implementation and perceived challenges. For CECAN self-organised teams, in a networked context requiring new participation strategies, working on case studies following APM principles provided a structured approach to a different style of management of evaluation and research-related tasks. Teams perceived positive attributes that are also referenced in previous studies about agile methods, including easy adoption and relation to project success (Serrador and Pinto, 2015), as well as improved teamwork through the focus on human and social factors (Dybå and Dingsøyr, 2008). Several interviewees highlighted the key role of the Scrum Master as facilitator but showed less agreement in relation to new concepts when applied to scientific activity such as “sprint development”, or the importance of small and regular releases of research outputs, when applied to scientific activity.

Studies on agile management have demonstrated the benefits to be gained with respect to fostering trust and cohesion in teams (McHugh et al., 2012). Empirical evidence points to a correlation with differing levels of shared leadership, team orientation, cross-functionality, internal learning processes and team autonomy (Moe et al., 2009; Stettina and Heijstek, 2011). This seems to be the case as well in the specific research context studied at CECAN, and also when contrasted with perspectives from other researchers who are familiar with agile methods. Some of the limitations of agile methods addressed by academic literature are also present in this case, such as the difficulties experienced by certain individuals or personality types in properly integrating into agile teams (Whitworth and Biddle, 2007). As well as the constraints perceived as inherent to the tradition of academic institutions and the lack of new management practices in scientific activity (Pope-Ruark, 2017), or difficulties in adapting to digital tools by senior researchers, some other complexities of adopting agile methods for research were evident. For instance, the timeframes for developing intellectual activity, and the motivation for doing so, can vary significantly depending on the type of project. Also, some researchers held the view that there should be a balance between prescriptive and adaptable formulas for this type of dynamic management.

In relation to specific tools, only a relative minority of researchers were active on the Trello platform, despite the entire organisation having full access to all the boards. This unequal distribution of participation via the digital Kanban board seems to represent a typical “90/9/1 principle” or “power law” (Nielsen, 2006), usually present in online communities of peer production, where the fact that a large percentage of people do not contribute does not necessarily constitute a problem or put at risk the achievement of common goals (Fuster Morell, 2014). In this sense, for a number of researchers, the proliferation of Trello boards represented an organisational challenge in terms of managing the tasks in progress and staying on top of all the boards, once several boards were in active use, which coincides with the findings of other studies about the adoption of digital Kanban boards for knowledge management in distributed organisations (McLean and Canham, 2018).

Lessons learned from this case study point to the need to reconsider the suitability of the scrum framework as the best agile approach for distributed research management. Future studies should explore if more open interpretations of APM practices (which for example focus on the regular but less structured updating of tasks via Kanban boards) could be more successfully adopted in this context, or if on the contrary, additional scrum practices (such as regular “standups” in short periods, or retrospective meetings) could improve the adaptation of APM principles and practices adapted to research activity. Another relevant issue emanating from this exploratory study relates to whether the adoption of professional agile facilitation (by experts in scrum or other agile practices and not researchers) is important and should be addressed with a comparative focus in future cases. As one of its main limitations, this study did not gather data that could compare adoption in such terms. Finally, in relation to the critical factor of remote, distributed research teamwork, another line of inquiry should address how agile practices could be used effectively in fully allocated science teams, where sharing the same physical space could benefit from the use of offline Kanban boards, as opposed to digital ones.

## Declarations

### Author contribution statement

Enric Senabre Hidalgo: Analyzed and interpreted the data; Contributed reagents, materials, analysis tools or data; Wrote the paper.

### Funding statement

This work was supported by CECAN and University of Surrey under a Fellowship grant agreement.

### Competing interest statement

The authors declare no conflict of interest.

### Additional information

No additional information is available for this paper.
